# “Effects of Redox Status on Immediate Hypericin-Mediated Photodynamic Therapy in Human Glioblastoma T98G Cell Line”

**DOI:** 10.1021/acsomega.4c08553

**Published:** 2024-12-28

**Authors:** Camila Aparecida Errerias Fernandes Cardinali, Camila Fabiano de Freitas, Renato Sonchini Gonçalves, Flavia Amanda Pedroso de Morais, Juliana Nunes de Lima Martins, Yandara Akamine Martins, Jurandir Fernando Comar, Patrícia de Souza Bonfim-Mendonça, André Luiz Tessaro, Elza Kimura, Wilker Caetano, Noboru Hioka, Kellen Brunaldi, Maria Ida Ravanelli

**Affiliations:** † Departament of Physiological Sciences, State University of Maringa, Maringa, Parana 87020-900, Brazil; ‡ Department of Chemistry, Federal University of Santa Catarina (UFSC), Florianópolis, Santa Catarina 88040-380, Brazil; § Departament of Chemistry, State University of Maringa, Maringa, Parana 87020-900, Brazil; ∥ Departament of Biochemistry, State University of Maringa, Maringa, Parana 87020-900, Brazil; ⊥ Department of Clinical Analysis and Biomedicine, State University of Maringa, Maringa, Parana 86812-460, Brazil; # Chemistry Graduation (COLIQ), Federal Technological University of Parana, Apucarana, Parana 86800-000, Brazil; ¶ Department of Pharmacy and Pharmacology, State University of Maringa, Maringa, Parana 87020-900, Brazil

## Abstract

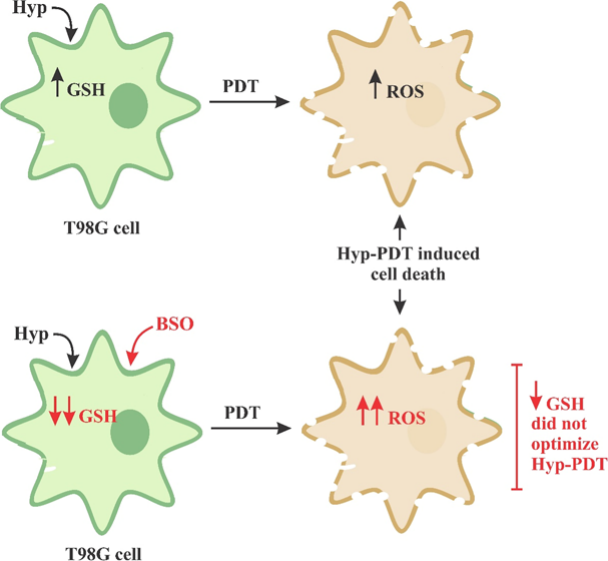

Glioblastoma Multiforme (GBM) is one of the most aggressive types of brain tumor. GBM can modulate glutathione (GSH) levels and regulate cellular redox state, which can explain its high resistance to chemotherapeutic agents. Photodynamic therapy (PDT) is a selective, nontoxic, and minimally invasive treatment approved for many types of cancer. PDT leads to cell death mainly by promoting the generation of reactive oxygen species (ROS). Thus, in the current study, PDT with the photosensitizer hypericin (Hyp), formulated in mixed 1,2-dipalmitoyl-*sn*-glycero-3-phosphocholine (DPPC)/biotinylated-pluronic F127 (F127–B) liposomes, in combination with the GSH synthesis inhibitor buthionine sulfoximine (BSO) were tested against T98G cell line of human glioblastoma. The mixed liposome was effective in delivering Hyp to the cells, leading to a dose relationship between Hyp and ROS levels. BSO potentiated Hyp cell uptake, decreased GSH levels regardless of Hyp concentration, and intensified ROS generation for 1.00 and 5.00 μmol L^–1^ Hyp. Nonetheless, cell death was more pronounced in the groups not treated with BSO, indicating that reduced GSH levels are not a decisive factor in achieving the PDT effects of Hyp. In conclusion, the mixed DPPC/F127–B liposomes were effective as a delivery system for Hyp. However, the combination of BSO and Hyp was not capable of optimizing PDT against T98G cells.

## Introduction

Glioblastoma multiforme (GBM) is a grade IV brain tumor, considered one of the most aggressive and challenging types of tumors.^1−3^ GBM treatment consists of surgical removal followed by chemotherapy and radiotherapy. Nonetheless, life expectancy is approximately 18 months.^4^ The impossibility of total tumor resection and difficult access to drugs to the brain contribute to the unfavorable prognosis and failure of therapeutic interventions.^5,6^ Furthermore, chemoresistance imposes major problems in the treatment of GBM. Elevated levels of glutathione (GSH), which protects cancerous cells from death by oxidative stress, have been proposed as one of the mechanisms related to the GBM resistance to drugs.^7^ GBM presents a high rate of recurrence, mainly in the brain tissue adjacent to the resection cavity of the tumor.

Photodynamic therapy (PDT) has been proposed as a selective adjunctive treatment to be administered in situ during surgical removal of residual neoplastic cells in marginal tissues.^8^ Therefore, it might be a promising alternative treatment against GBM. PDT is minimally invasive, nontoxic for healthy tissues, and selective against cancer cells. For PDT to occur it is necessary the presence of a photosensitizer (PS), light, and oxygen. PS, after excited by visible light, reacts with molecular oxygen (^3^O_2_) to produce reactive oxygen species (ROS) and especially singlet oxygen (^1^O_2_), which are responsible for cell death by necrosis or apoptosis.^9,10^

Hypericin (Hyp) is a promising PS due to its high quantum yield of singlet oxygen (ϕ_Δ_^1^O_2_),^10,11^ low cytotoxicity, and ability to generate ROS and induce cell death under photostimulation.^9,12,13^ Additionally, Hyp fluorescence properties can aid in the surgical resection of tumors.^14^ Nonetheless, Hyp has a lipophilic nature and forms insoluble aggregates in an aqueous environment, making in vivo administration troublesome.^15^

Liposomes containing polymers covalently functionalized with biotin have been used to improve the delivery of lipophilic PS to tumor cells.^16−18^ These nanostructures carry both hydrophilic and lipophilic drugs and are biocompatible, biodegradable, and can be modified according to the required application (surface characteristics, lipid composition, hydrodynamic diameter, and lamellarity).^19^ Polymer modifications, like biotin-functionalized liposomes, enhance properties such as micellization, drug entrapment, and targeting. These are used in treating cancer, infections, wound healing, cell regeneration, and tissue engineering.^20^

This study aimed to evaluate whether Hyp-mediated PDT, utilizing Hyp encapsulated in mixed liposomes composed of 1,2-dipalmitoyl-*sn*-glycero-3-phosphocholine (DPPC) and biotinylated pluronic F127 (F127–B), combined with the GSH synthesis inhibitor buthionine sulfoximine (BSO), could modulate endogenous GSH generation, thereby affecting ROS levels and enhancing PDT efficacy.

## Methods

### Preparation of Mixed Liposomes of DPPC and Biotinylated F127 with Hypericin Formulations

1,2-Dipalmitoyl-*sn*-glycero-3-phosphocholine (DPPC), F-127 pluronic triblock copolymers and Biotin were purchased from Sigma, USA. Biotinylated-F127 (F127–B) was synthesized, as previously described, and their purities were demonstrated by ^1^H NMR.^21^ Hyp (1,3,4,6,8,13-hexahydroxy-10,11-dimethylphenanthro [1,10,9, 8-opqra] perylene-7,14-dione, C_30_H_16_O_8_, 98% purity) was supplied by the Photodynamic Systems Research Group (NUPESF), Department of Chemistry, State University of Maringá. DPPC and F127–B (0.10% w/v of F127–B in DPPC) were used to prepare mixed unilamellar liposomes.^22^ Hyp was actively incorporated into the liposomes using the thin-film hydration method.^23^ The film was hydrated with Dulbeccós phosphate-buffered saline (DPBS, Gibco, USA) and sonicated at 40–45 °C in an ultrasonic bath (Cristófoli Biosafety Equipment) for 30 min, following the previous methodology.^18,24^

### Liposome Characterization

After the incorporation of Hyp, spectroscopic analyses of electronic absorption (UV–vis, Beckman Coulter DU 800, USA) and fluorescence emission spectroscopy (Cary-Eclipse) were conducted. The mean hydrodynamic diameter and polydispersity index (PI) were determined by dynamic light scattering (DLS) (NanoPlus). The morphology of the biofunctionalized lipid-coated formulations containing Hyp was evaluated using transmission electron microscopy JEOL JEM-1400 (Jeol Ltd., Japan). The samples were prepared with trehalose added as a cryoprotectant ([Tre] = 3.0 × 10^–4^ mol L^–1^). These samples were diluted 100-fold in PBS, and a drop was placed onto Formvar-coated copper grids. To enhance image resolution, the grids were quickly submerged in liquid nitrogen and freeze-dried for 30 min^25^ The encapsulation efficiency (EE %) and drug loading were quantified by centrifuging each sample through Microcon-10 kDa centrifugal filters. The formulations were centrifuged at 13,000 rpm for 30 min, and the free Hyp was quantified by fluorescence emission. The values were calculated based on eqs 1 and (2)
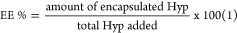

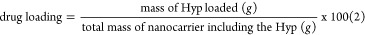


Light source and calculation of the absorbed light power (*P*_Abs_)

To calculate the absorbed power (*P*_Abs_) and the number of photons absorbed (*N*_Abs_) a spectroradiometer by Ocean Optics model USB2000+ was used. The calculations of *P*_Abs_ and *N*_Abs_ were performed following the previous methodology.^26^ The *P*_Abs_ is the product between the total power emitted by the source and the fraction of light absorbed by Hyp. For a polychromatic light source, *P*_Abs_ is defined by eq 1
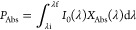
1In which, λ_i_ and λ_f_ are the starting and ending wavelengths of the LED irradiation spectrum, *I*_0_ is the absolute irradiance emitted by the LEDs and *X*_Abs_ is the fraction of light absorbed by Hyp. *X*_Abs_ is given by eq 2 for polychromatic sources
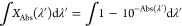
2

*N*_Abs_ can be obtained by eq 3
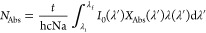
3In which,  is the Planck constant (6.626 × 10^–34^, in J s^–1^), *c* is the speed of light (*c* = 2.997 × 10^8^ ms^–1^) and Na is the Avogadro constant (Na: 6.022 × 10^23^ mol^–1^), which represents an equation in the number of moles of photons emitted (equivalent to Einstein).

### Cell Culture and Treatment Protocols for Hypericin in Glioblastoma T98G Cells

**Figure 1:**
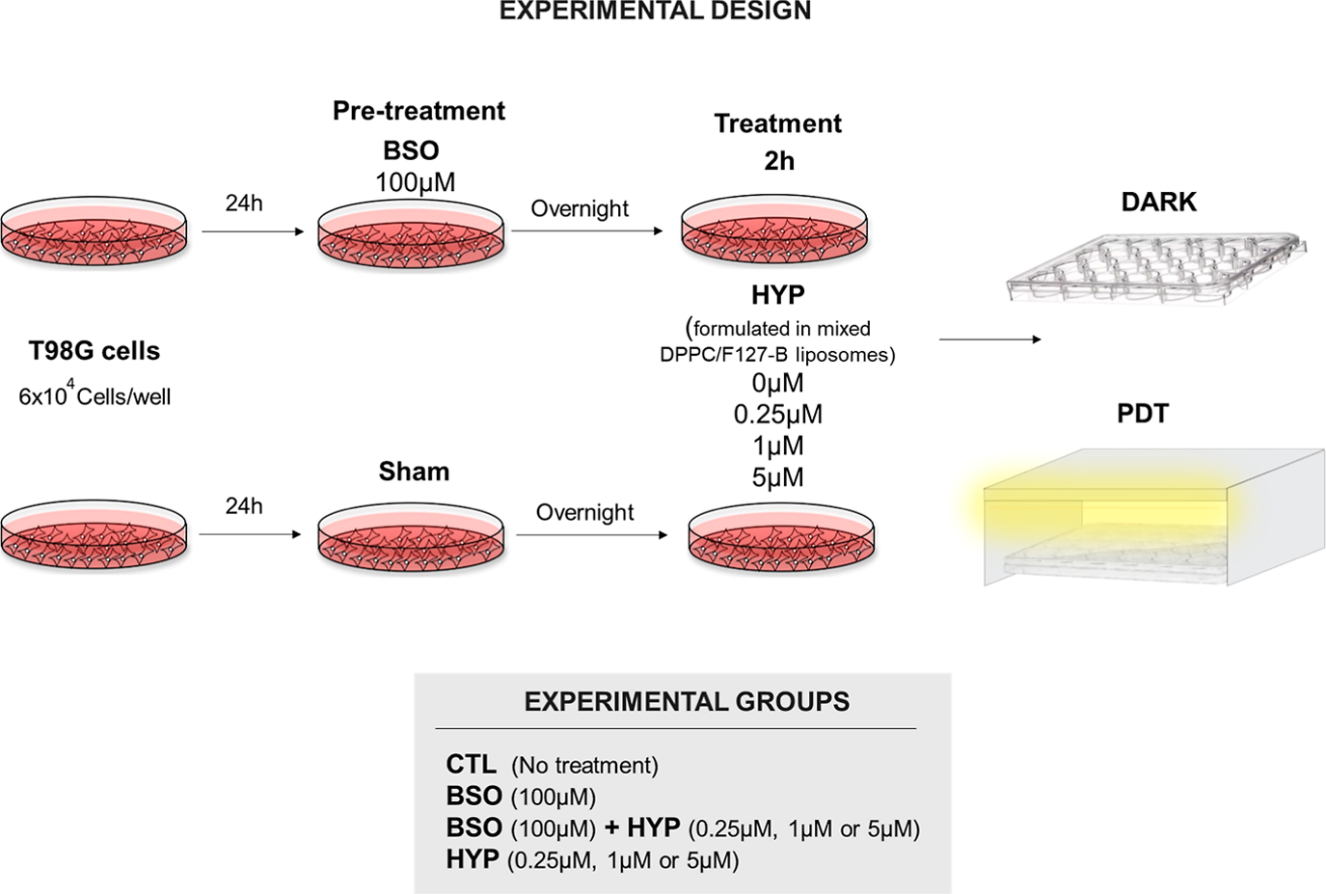
Experimental design and groups. BSO = l-buthionine [S,R]-sulfoximine, HYP = Hypericin, DPPC = 1,2-dipalmitoyl-*sn*-glycero-3-phosphocholine, F127–B = biotinylated-pluronic F127, PDT = Photodynamic therapy, CTL = control group.

Human glioblastoma T98G cells (ATCC CRL-1690) were cultured in Dulbecco’s Modified Eagle’s Medium (DMEM, Gibco, USA) with 10% fetal bovine serum (FBS, Gibco, USA) and 1% penicillin–streptomycin (Sigma, USA) at 37 °C in a 5% CO2 humidified incubator. Before treatment, cells were preincubated overnight (16 h) with 100 μM l-buthionine [S,R]-sulfoximine (BSO; Sigma, USA) in DMEM, or with DMEM alone as a control, as shown in the experimental design in Figure 1. Following pretreatment, cells were incubated for 2 h with hypericin (Hyp) encapsulated in mixed DPPC–F127–B liposomes at concentrations of 0.25, 1.00, and 5.00 μmol/L. The concentrations of hypericin used were selected from a dose–response experiment shown in the Supporting Information (Figure 1S). Experiments used freshly prepared Hyp/liposome solutions.

### Photodynamic Therapy

Cells were submitted to 20 min of PDT (20 mW cm^–2^) using a homemade device containing 66 units of warm white LEDs (450 to 750 nm, 20 mW/cm^2^). The lighting system used in the present study is represented in Figure 2S of the Supporting Information

### Cellular Uptake of Hypericin Measured by Flow Cytometry

T98G cells were seeded at a density of 3 × 10^5^ cells/mL in 6-well culture plates. After trypsinization (0.05% trypsin–EDTA, Gibco, USA), the cells were centrifuged at 2500 rpm for 8 min at 4 °C. The resulting cell pellet was resuspended in 200 μL of MOPS buffer (3-morpholinopropane-1-sulfonic acid, Sigma, USA, pH 7.4). Hypericin-labeled cells were then analyzed using a flow cytometer (BD FACS Calibur, San Jose, CA, USA).

### Cell Imaging Using Confocal Fluorescence Microscopy

Cells were seeded at a density of 6 × 10^4^ cells/well in a 24-well culture plate with a 13 mm coverslip bottom (Knittel Glass). Imaging was performed using a confocal fluorescence microscope (λ_ex_ 488 and λ_em_ 543 nm) with a 40× objective.

### Measurement of Glutathione Concentrations

Cells were seeded at a density of 1.2 × 10^5^ cells/well in 6-well culture plates. Following trypsinization (0.05% trypsin–EDTA), cells were centrifuged at 2500 rpm for 8 min at 4 °C. The cell pellet was resuspended in 200 μL of MOPS buffer (pH 7.4). Total protein concentration was measured using the Folin phenol reagent^27^ with 100 μL of the cell suspension. To determine glutathione (GSH) concentration, 100 μL of the cell suspension was deproteinized with 50 μL of trichloroacetic acid (TCA) and centrifuged at 9800 rpm for 3 min at 4 °C. The GSH concentration in the supernatant was measured using the *o*-phthalaldehyde (OPT) method (Sigma, USA)^28^ adapted for a fluorescence microplate reader (FlexStation 3, Molecular Devices-λ_ex_350/λ_em_420 nm). GSH levels were expressed as μmol GSH·(mg protein)^−1^.

### Measurement of Reactive Oxygen Species Using DCFH-DA

Cells were seeded at a density of 6 × 10^4^ cells/well and incubated with 15 μmol/L of the fluorescent probe DCFH-DA for 30 min (2,7-dichlorodihydrofluorescein diacetate, Sigma, USA).^29^ The fluorescence intensity of DCFH-DA was measured using a microplate reader (λ_ex_504/λ_em_529 nm).

### Cell Viability with Trypan Blue

Cells were seeded at a density of 6 × 10^4^ cells/well in 96-well culture plates and subjected to 20 min of PDT at 20 mW/cm^2^. Following PDT, cells were trypsinized (0.05% trypsin–EDTA), centrifuged (2500 rpm, 8 min, 4 °C), and resuspended in 0.4% trypan blue in DMEM at a 1:1 (v/v) ratio. The same procedure was performed for dark control wells, which were not subjected to PDT. Viable cell counts were determined in triplicate for each well and expressed as a percentage relative to control wells, where cell viability was set at 100%.

### Statistical Analysis

Data were submitted to the Shapiro–Wilk test for normality. Parametric data were analyzed using one-way or two-way ANOVA, followed by a post hoc test (Tukey’s HSD) for multiple comparisons. Nonparametric data were analyzed using the Kruskal–Wallis test with post hoc comparisons. Data are presented as mean ± standard error of the mean (SEM). Statistical significance was considered at the 95% confidence level. All statistical analyses were conducted using GraphPad Prism software version 8.0.

## Results and Discussion

### Light Dose and Characterization of Hypericin Encapsulation in Mixed Liposomes of DPPC and Biotinylated-F127

One of the main obstacles to the treatment of brain tumors is drug delivery. Rarely does a drug in an aqueous solution or an organic medium reach the targeted tissue in adequate concentrations. Thus, liposomal drug carrier systems have been used as a promising approach to enhance the solubility of both hydrophilic and hydrophobic drugs. Liposomes provide several advantages compared to conventional formulations based on free drugs, as evidenced by their high biocompatibility, biodegradability, lower therapeutic doses and toxicity, and the capacity to combine therapies.^30^

Tumor cells overexpress receptors to increase their metabolism.^31,32^ Therefore, receptor agonists, such as biotin, have been employed to enhance drug selectivity and accumulation in neoplastic cells. In recurrent GBM, the avidin–biotin technology yielded great results in patient survival.^33^ Hyp is considered a theranostic PS, that is, it can be applied both in PDT and in the detection of malignant cells.^14^ However, in an aqueous environment, the elevated tendency of Hyp to self-aggregate considerably reduces the light absorption and its emission capacity, which harms the generation of ^1^O_2_. In this regard, liposomal drug delivery systems play an important role in maintaining Hyp as a monomer to increase its stability, bioavailability, and consequently its photodynamic and theranostic efficiency.^34^

**Figure 2:**
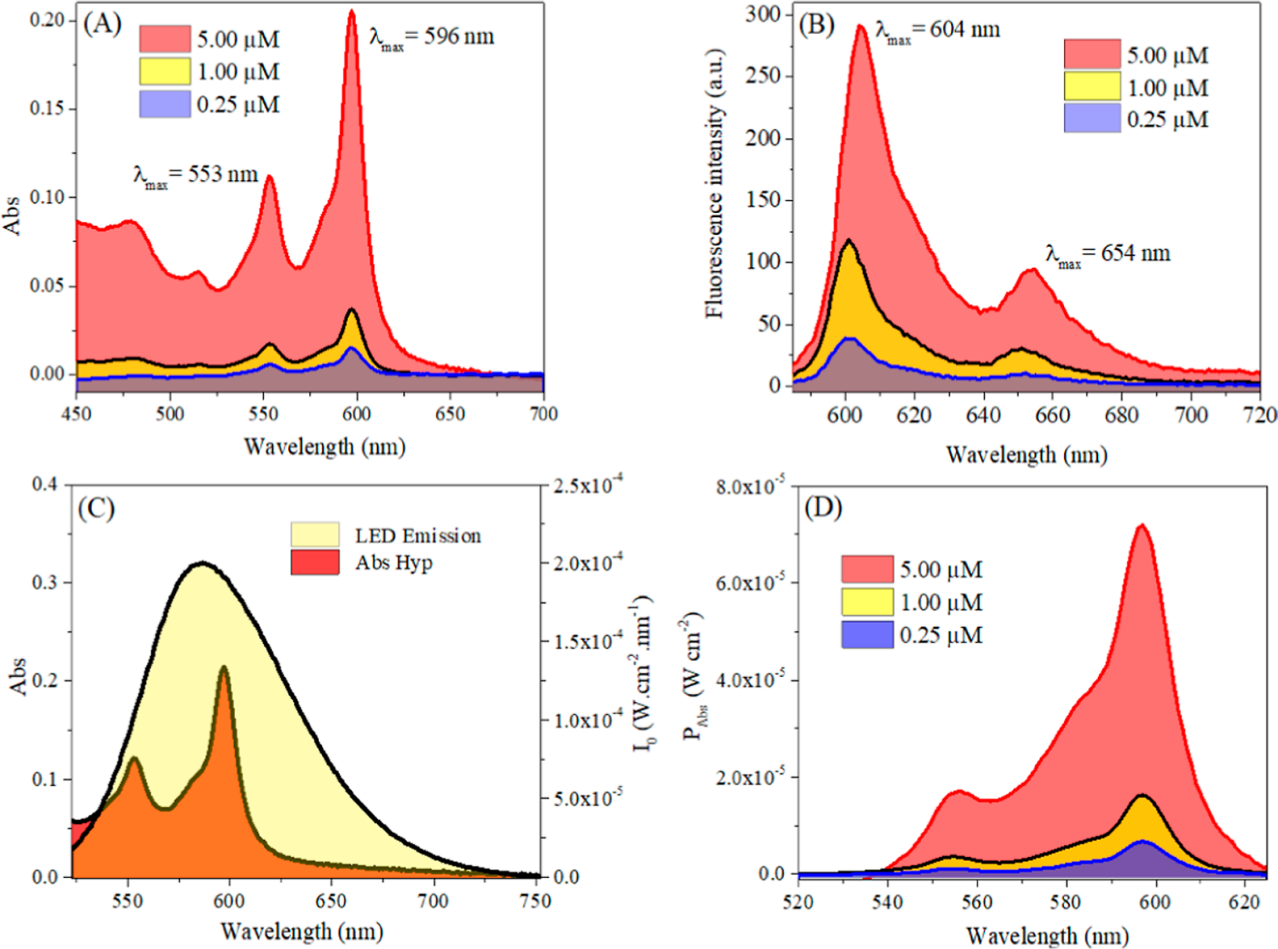
(A) Absorption spectra of the Hyp DPPC/F127–B mixed liposomes in DPBS buffer. (B) Emission fluorescence spectra of Hyp DPPC/F127–B mixed liposomes in DPBS, _exc_ = 554 nm. (C) Overlapping of the Hyp absorption spectra and emission of the light source. (D) Absorptive potency (*P*_Abs_) of Hyp encapsulated in mixed liposomes 0.10% F127/DPPC; [DPPC] = 1.5 × 10^–3^ mol L^–1^.

Therefore, the first aim of this work was to obtain liposomal preparation containing Hyp in the monomer form that could be excited in a PDT setting. Representative spectra of the electronic absorption and the fluorescence emission of Hyp encapsulated in mixed DPPC/F127–B liposomes are depicted in Figure 2A,B, respectively. Figure 2A shows a well-defined spectral profile characteristic of the monomeric Hyp: two main absorption peaks, one with maximum wavelength (λ_max_) at 553 nm and another one at 596 nm of higher intensity.^35^ The presence of Hyp monomers in the liposomal preparation was further corroborated by the analysis of the fluorescence emission spectra presented in Figure 2B. The two maximum emission wavelengths at 604 and 654 nm indicated the lack of Hyp aggregates in,^36^ which otherwise would have led to self-quenching of energy and reduction of radioactive transitions.^37^

To achieve a high PDT efficiency, the PS absorption spectrum and the emission of the light source must overlap, as evidenced in Figure 2C. Furthermore, as expected, Hyp at higher concentrations was able to absorb a greater amount of radiation as indicated by absorptive potency (*P*_Abs_) data shown in Figure 2D. Altogether, the results presented in Figure 2 indicate that monomers of Hyp, for all concentrations of Hyp tested, were properly incorporated into the lipid bilayer of mixed DPPC/F128–B liposomes and excited by the light source.

The enhanced permeability and retention (EPR) Effect allows the accumulation of high-molecular weight drugs in tissues with increased permeability such as tumors.^32,38^ To achieve the EPR a drug delivery system must present a diameter less than 200 nm. As indicated in Table 1 the average distribution size of the hydrodynamic diameter (DH) of the DPPC/F127–B liposomes with Hyp was not higher than 162.5 nm. Furthermore, the PI of the liposomes was below 0.3, indicating the uniformity of the liposome preparation. Therefore, these results suggest that liposomes containing Hyp (actively incorporated by sonication using the thin film methodology)^18,24^ presented the expected structural features required for the delivery of Hyp to cancer tissues. Representative images of DLS analyses to evaluate the average size of liposomal vesicles based on the number, distribution intensity, and volume of all vesicles produced are shown in Figure 3S of the Supporting Information

**Table 1:** Hydrodynamic Diameter (DH) and Polydispersity Index (PI) of Hyp Formulated in Mixed DPPC/F127-B Liposomes^a^

Hyp (μmol·L^–1^)	DH (nm)	PI
0	138.4 ± 16.8	0.258 ± 0.015
0.25	140.0 ± 23.9	0.281 ± 0.008
1.00	141.4 ± 10.8	0.269 ± 0.005
5.00	162.5 ± 39.4	0.280 ± 0.016

a (*n* = 6). The values are presented as mean ± SEM.

**Figure 3:**
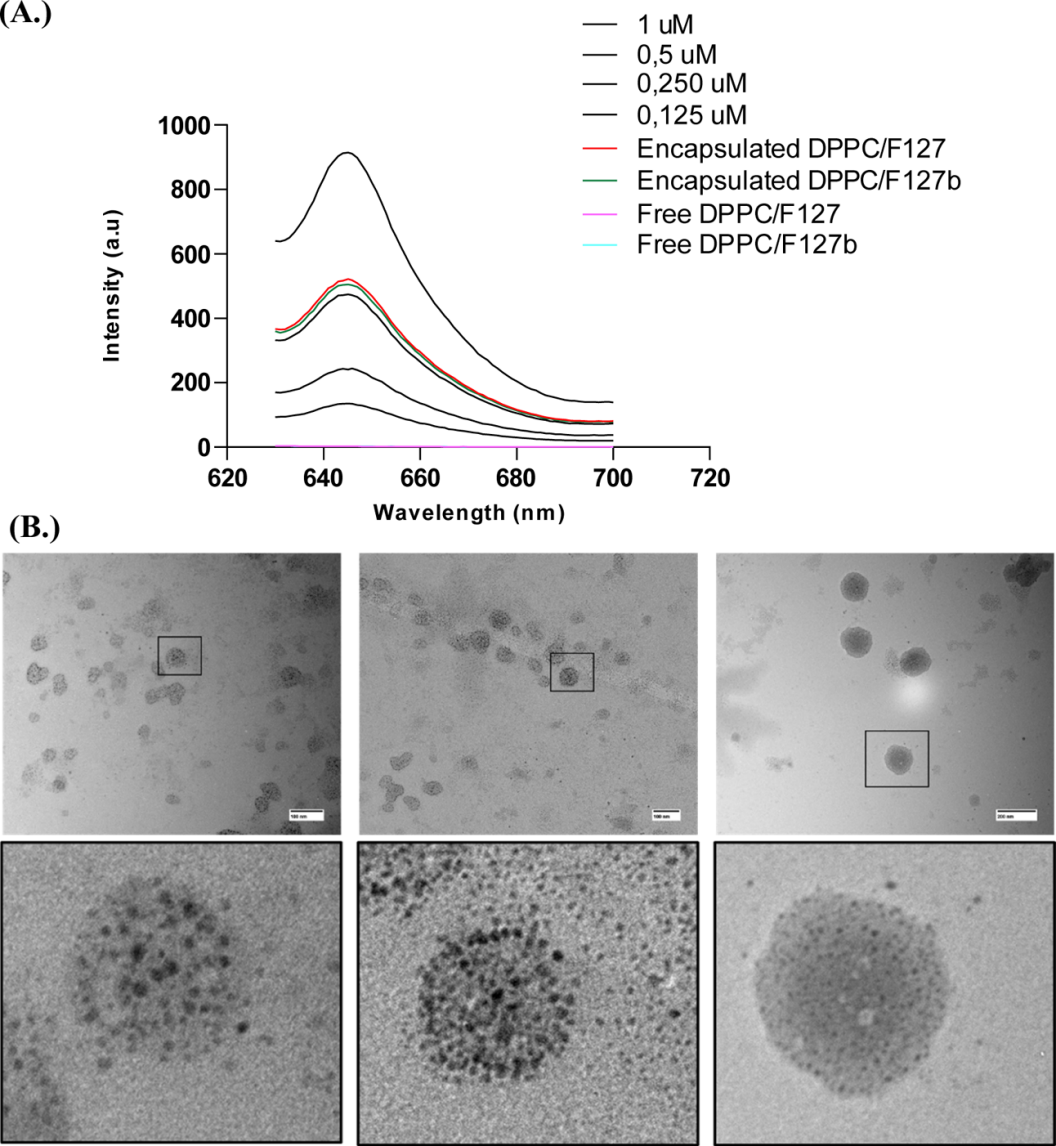
(A) The EE % and (B). Representation of transmission electron microscopy images for Hyp (5.00 μmol L^–1^) nanoformulation in DPPC liposomes with biotin-linked Pluronic F127.

Additionally, the morphology of Hyp formulation, as well as the EE % and drug loading, were quantified. The EE % and the drug loading determined for Hyp at 5.00 μmol L^–1^ was, 86.4% (±0.1) and 0.16%, respectively, highlighting once again the superb properties of the liposomal as a drug delivery system (Figure 3A). The %EE is higher than the observed for DSPC liposomes^39^ and in line with the efficiency obtained in DPPC liposomes.^40^ The morphology of the nanoparticles was verified through TEM analysis (Figure 3B), which displayed dispersed spherical structures. The size of the vesicles agrees with those obtained from DLS assuring the successful interaction between biotinylated F127, Hyp, and DPPC, preserving size uniformity and spherical shape.

### Hypericin Uptake and Localization in T98G Cells

**Figure 4:**
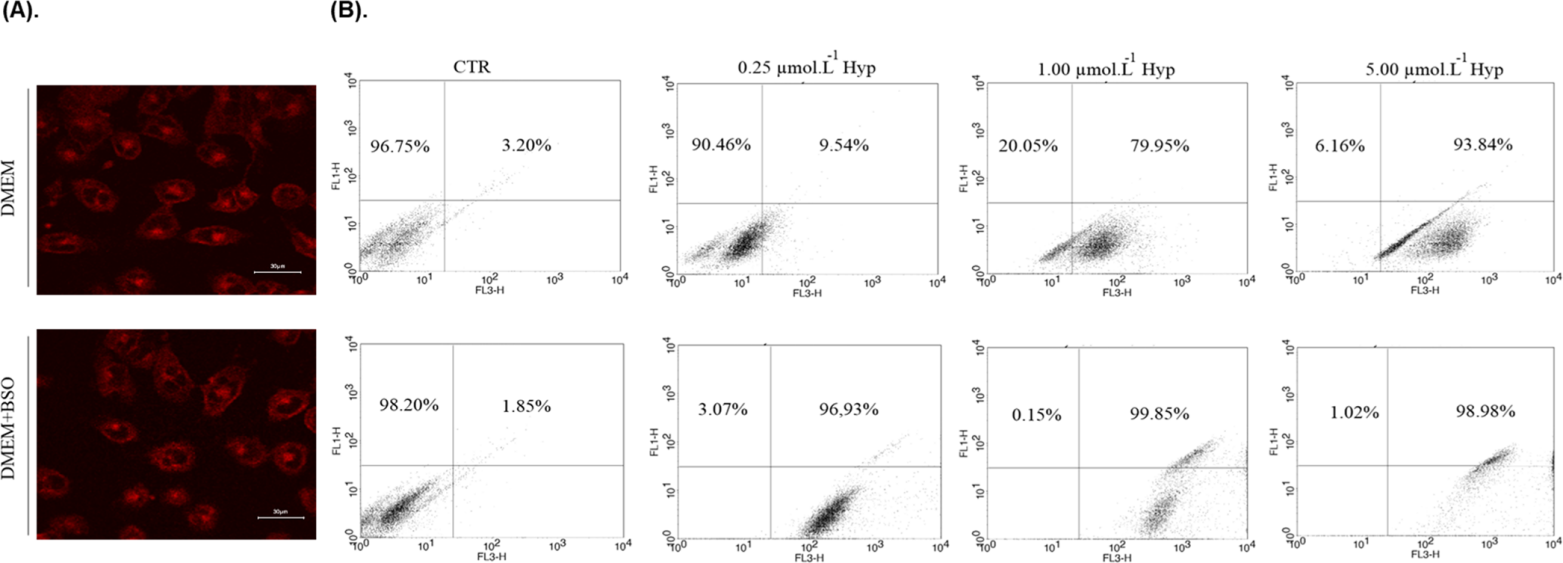
(A) Confocal fluorescence images of Hyp (5 μmol L^–1^) localization in T98G cells using a 40× objective. (B) Hyp uptake by T98G cells measured by flow cytometry. T98G cells were pretreated with BSO (100 μmol L^–1^) or DMEM for 16 h followed by 2 h treatment with Hyp (0.25, 1, and 5 μmol L^–1^) encapsulated in DPPC/F127–B mixed liposomes. Confocal fluorescence images at 5 μmol L^–1^ of Hyp DPPC/F127–B mixed liposomes.

The efficiency of a PS is strongly related to its distribution and accumulation in cell compartments.^8^ Therefore, flow cytometry in combination with confocal fluorescence microscopy was used to monitor the delivery and cell uptake of Hyp. As shown in Figure 4A, confocal fluorescence images indicate that Hyp was properly delivered by the DPPC/F127–B mixed liposomes and accumulated preferentially in the plasma and nuclear membranes of T98G cells, as previously reported.^13^

Although flow cytometry does not allow quantifying the cellular content of Hyp, BSO pretreatment produced an increase in the cell population labeled with Hyp, regardless of Hyp concentration, in comparison to the control group (Figure 4B). This result may be explained, at least in part, by an effect of BSO on the membrane permeability to Hyp, considering that BSO (400 μmol L^–1^, 24 h) reduces the concentration of membrane sphingomyelin and increases the membrane fluidity of HepG2 cells.^41^

**Figure 5:**
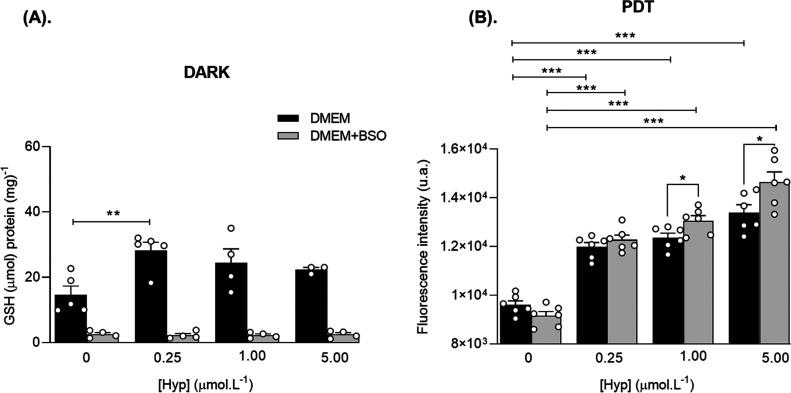
(A) Total GSH levels of T98G cells pretreated with BSO (100 μmol L^–1^,16 h) or DMEM followed by treatment with Hyp (2 h) in the dark. (B). ROS levels evaluated by DCF after PDT (20 min, 20 mW/cm^2^) of T98G cells pretreated with BSO (100 μmol L^–1^,16 h) or DMEM followed by treatment with Hyp (2 h). Results are presented as mean ± SEM (*n* = 3–6).**p < 0.01, ***p < 0.001.

In addition, in the absence of BSO, the cellular efflux of Hyp might have been intensified by the elevated GSH levels (Figure 5A). Hyp is a substrate of the glutathione-*S*-transferase (GST), a family of detoxifying enzymes implicated in the development of resistance toward chemotherapy agents and that is commonly upregulated in tumors.^42^ GST catalyzes the binding of xenobiotics to GSH with the expulsion of the GSH/xenobiotic complex by a cell membrane-bound GS-X pump.^43^

### Status Redox

ROS and antioxidant mechanisms are generated by all aerobic cells. Within physiological levels, ROS are important signaling molecules.^44^ On the other hand, elevated ROS are involved in tumor initiation, progression, and regression. In response to ROS augmentation, cancer cells increase the levels of GSH and other endogenous antioxidant molecules to protect themselves from ROS deleterious effects.^45,46^

GSH is a tripeptide known to be the most abundant low molecular-weight thiol synthesized by cells. GSH plays an important role in protecting cells against oxidative stress and in the elimination of toxic electrophilic xenobiotics.^43,47^ Additionally, changes in the redox balance through modulation of GSH levels appear to be involved in the mechanism of GBM chemoresistance.^7,48−50^ Thus, the current study hypothesized that the depletion of GSH induced by BSO would potentiate Hyp-mediated PDT against GBM.

Total GSH levels of T98G cells in the dark are illustrated in Figure 5A. Hyp, in the absence of BSO, produced a marked increase in GSH levels of 91.9% after 2 h of incubation with the lowest concentration of 0.25 μmol L^–1^ (*p* = 0.0085). However, with the rise in Hyp concentration, there was a slight reduction in GSH levels (20.8% increase with 5.00 μmol L^–1^ of Hyp), suggesting the formation of Hyp-GSH complexes as discussed previously. Therefore, these results indicate that Hyp per se is capable of modulating GSH levels, which may impact cellular protection against oxidative stress. As expected, the specific inhibitor of the enzyme γ-glutamylcysteine synthetase BSO, reduced GSH levels, abolishing the Hyp rising effect on GSH levels. No difference in GSH concentration was found between the control and Hyp-BSO groups.

The next step of this work was to evaluate the generation of ROS by Hyp, a mandatory feature of the PDT. Hyp is one of the most effective natural PS due to its extensive double-bound conjugated system. Upon light absorption, Hyp in the ground state S_o_ is converted to an excited singlet state S_1_, which may undergo intersystem crossing to form a more stable triplet excited state T_1_. Hyp in the triplet state can either decay radiationlessly to S_o_ or transfer its energy to molecular oxygen (O_2_), leading to the formation of singlet oxygen (^1^O_2_). Subsequently, the singlet oxygen forms peroxide.^51^ Hyp can also generate superoxide free radicals under the irradiation of visible light and in the presence of oxygen.^52^

Figure 5B depicts the cellular levels of ROS after Hyp PDT (20 min, 20 mW/cm^2^) in cells Hyp-BSO. ROS production was indirectly measured by the DCFH-DA method. Cytoplasmic esterases catalyze the cleavage of DCFH-DA into H2DCF, both nonfluorescent cell impermeable molecules. When H_2_DCF is oxidized by peroxides, the fluorescent product DCF is formed.^53^

All groups treated with Hyp exhibited increased ROS levels compared to their respective controls (i.e., with and without pretreatment with BSO) after PDT (Figure 5B). For the Hyp groups, increases in ROS levels of 24.68% (F(3.40) = 4.26, *p* < 0.001), 28.68% (F(3.40) = 4.26, *p* < 0.001), and 39.43% (F(3.40) = 4.26, *p* < 0.001) were observed for concentrations of 0.25, 1.00, and 5.00 μmol L^–1^ compared to the CTL, respectively (Figure 5B). For the BSO pretreated groups, increases of 34.12% (F(3.40) = 4.26, *p* < 0.001), 42.6% (F(3.40) = 4.26, *p* < 0.001), and 60.04% (F(3.40) = 4.26, *p* < 0.001) were observed for concentrations of 0.25, 1.00, and 5.00 μmol L^–1^ compared to CTL BSO, respectively (Figure 5B). Additionally, ROS levels increased by 5.6% and 9.4% for Hyp-BSO concentrations of 1.00 and 5.00 μmol L^–1^, respectively, compared to Hyp alone (*p* < 0.05). Interestingly, no difference in ROS generation was observed for the dose of 0.25 μmol L^–1^, which had a higher population of cells marked, with Hyp-BSO (96.9%) vs (9.54%) Hyp (Figure 5B) and an increase in GSH levels (91.9%) (Figure 5A). In vitro studies suggested that the binding of Hyp to GSH, catalyzed by GST, suppresses the photophysical properties of Hyp, reducing ROS generation.^54^ This effect may have been mitigated by the higher concentrations of Hyp.

### Cell Viability with Trypan Blue

**Figure 6:**
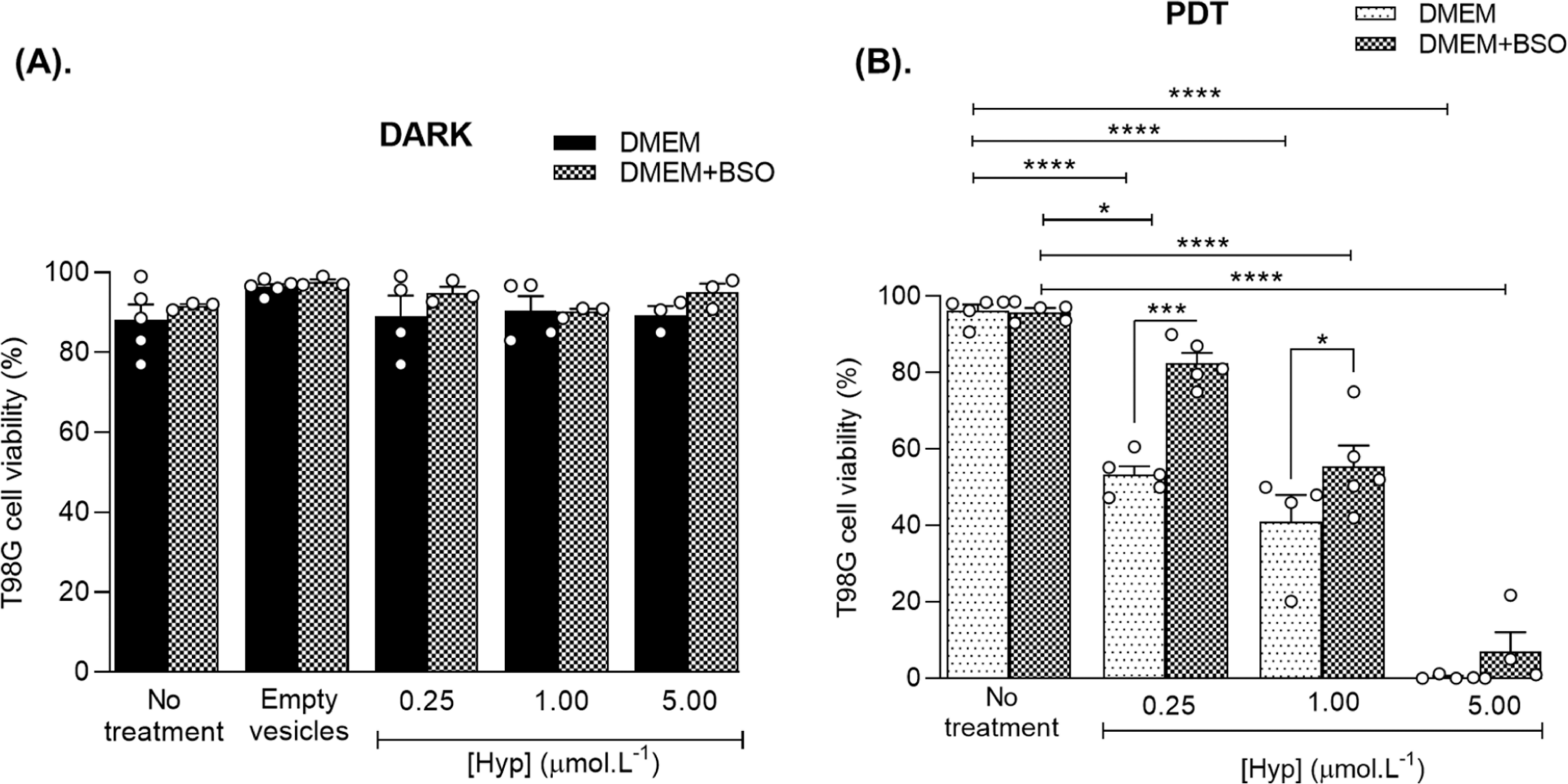
(A) Cell viability of T98G cells pretreated with BSO (100 μmol L^–1^, 16 h) or DMEM followed by treatment with Hyp or empty vesicles for 2 h in the dark and (B). After PDT (20 mW/cm^2^, 20 min) no treatment refers to cells cultivated in DMEM medium, in the absence and the presence of BSO. Results are presented as mean ± SEM (*n* = 3–5). *p < 0.05, **p < 0.01, ***p < 0.001.

Figure 6 shows cell viability assessed by Trypan blue in the dark and after PDT. In agreement with the literature,^55−57^ in the dark Hyp was not cytotoxic (Figure 6A). Conversely, Hyp PDT produced a remarkable dose-dependent reduction in cell viability (Figure 6B). For the Hyp groups, cell viability decreases by 43.1% (F(4.17) = 118.7, *p* < 0.001), 55.4% (F(4.17) = 118.7, *p* < 0.001), and 96.04% (F(4.17) = 118.7, *p* < 0.001) for concentrations of 0.25, 1.00, and 5.00 μmol L^–1^, respectively, compared to the CTL group (Figure 6B). For the BSO pretreated groups, cell viability decreases by 13.38% (F(3.15) = 93.14, *p* = 0.02), 40.3% (F(3.15) = 93.14, *p* < 0.001) and 89.02% (F(3.15) = 93.14, *p* < 0.001) for concentrations of 0.25, 1.00, and 5.00 μmol L^–1^, respectively, compared to the CTL BSO group (Figure 6B). Additionally, pretreatment with BSO reduced cell death by 54.8% (F(7,29) = 96.81, *p* < 0.001) and 35.26% (F(7,29) = 96.81, *p* = 0.011) for concentrations of 0.25 and 1 μmol L^–1^, respectively, after PDT(Figure 6B).

It is important to note that cell viability was assessed immediately after PDT, and cells were treated with Hyp for only 2 h. These immediate effects of Hyp PDT were observed at the microscope as a disintegration of the cell membrane, especially at higher concentrations of Hyp (data not shown). Furthermore, illumination in the absence of Hyp did not affect cell survival. In summary, these results confirm the effectiveness of DPPC/F127–B mixed liposomes as a delivery system for Hyp PDT.

Light activation of Hyp induces cytotoxicity through the generation of singlet oxygen and ROS.^58^ Additionally, Hyp can acidify the intracellular environment, which disrupts cell metabolism and can lead to cell death over time14,59Clique ou toque aqui para inserir o texto. Hyp PDT can trigger cell death by various mechanisms, including apoptosis and autophagy.^60−63^ Thus, we cannot exclude that the reduction in cell viability induced by Hyp PDT (Figure 6B) might be underestimated.

Our primary hypothesis was that BSO would potentiate Hyp PDT by influencing Hyp cell uptake and accumulation (Figure 3B), antioxidant defense (through reduction in GSH levels; Figure 5A), and ROS generation (Figure 5B). Contrary to our expectations, even though there was a reduction in the viability of cells submitted to BSO pretreatment and Hyp PDT, cell inactivation was more pronounced in the absence of BSO. These results suggest a potential neutralizing effect of BSO on Hyp PDT, or BSO-induced resistance to Hyp PDT, particularly evident at concentrations of 0.25 and 1.00 μmol L^–1^ of Hyp.

Nonetheless, we conclude that GSH levels are not a determining factor in the outcome of Hyp PDT. Resistance to Hyp PDT by some breast adenocarcinoma cell lines has been attributed to the enhanced expression of glutathione peroxidase 4 (GP-X4 pump),^48^ and possibly lower uptake of Hyp. Additionally, BSO is known to affect other antioxidant systems with crucial roles in ROS homeostasis (i.e., catalase inhibition).^58^ Finally, our results do not exclude the activation of compensatory pathways leading to restoring the redox balance and cell viability during the 16 h pretreatment with BSO.

Photoinduced oxidative membrane damage is considered a key step in PDT.^10,64^ However, due to the limited-time-action of the oxygen singlet in cells (short lifetime of 10–320 ns and diffusion to only 10 to 55 nm), PS molecules (and the oxygen singlet generated) must be located close or within the cell membrane.^65^ Accordingly, it is expected that the composition and physical properties of the cell membranes would indirectly influence the PDT efficiency. Thus, we speculate that the neutralizing effect of BSO against Hyp PDT may be the result of changes in Hyp interaction with the lipid bilayer.

Indeed, emission spectra of Hyp (monomer prevalence) vary according to the membrane lipid composition.^66^ Additionally, the amount of O_2_ and oxygen singlet presented in the lipid bilayer increases with the increase in fluidity.^67^ In this regard, the well-known rigidification effect of Hyp on cell membranes^59,68^ might oppose the generation of oxygen singlet, compromising PDT efficiency.

## Conclusion

In conclusion, the formulated Hyp encapsulated in mixed liposomes of biotinylated DPPC/F127 was effectively internalized by T98G cells, demonstrating no cytotoxicity in the dark. Following PDT, a concentration of 5.00 μmol L^–1^ of Hyp resulted in necrosis across nearly the entire cell population, indicating the potential of this delivery system as a promising adjunct in GBM treatment. However, although BSO pretreatment successfully reduced GSH levels and enhanced ROS production post-PDT, it did not induce immediate cell death, suggesting a limitation in the efficacy of Hyp under these conditions. Further research is needed to optimize this approach for in vivo applications.

## Limitations

This study has some limitations that should be considered. First, the use of a specific glioblastoma cell line, T98G, limits the generalization of the results, as other tumor cell lines might respond differently to the treatment. Additionally, only a few antioxidant parameters were evaluated, which does not allow for a complete understanding of the cellular redox status; more experiments would be necessary to better elucidate this balance. Our objective was to evaluate the immediate effect of PDT, so we did not consider the long-term effects of the drugs or the mechanisms of delayed cell death in a broader context.

## Supporting Information Available

Figure 01S shows the EC50 curve of hypericin in T98G cells (0.1, 0.25, 0.50, 1.00, 2.00, 5.00, and 10.00 μmol L^–1^) with an incubation time of 2 h. Figure 02S describes the lighting system used in the study. Figure 03S displays representative images from DLS analysis to evaluate the average size of liposomal vesicles based on intensity, volume, and number distribution for all vesicles produced (PDF)
