# Supplementary material for: “Effects of Redox Status on Immediate Hypericin-Mediated Photodynamic Therapy in Human Glioblastoma T98G Cell Line”
Source: ACS Omega. 2024 Dec 28;10(1):1100–9. doi: 10.1021/acsomega.4c08553 (PMC11740150; doi:10.1021/acsomega.4c08553)
Supplement: Supplementary file 1 — ao4c08553_si_001.pdf [file ao4c08553_si_001.pdf]

Supporting information:

"Effects of Redox Status on Immediate Hypericin-Mediated Photodynamic Therapy in Human Glioblastoma T98G Cell Line"

Camila Aparecida Errerias Fernandes Cardinali[1*], Camila Fabiano de Freitas[2*], Renato Sonchini Gonçalves[3], Flavia Amanda Pedroso de Morais[3], Juliana Nunes de Lima Martins[4], Yandara Akamine Martins[1], Jurandir Fernando Comar[4], Patrícia de Souza Bonfim-Mendonça[5], André Luiz Tessaro[6], Elza Kimura[7], Wilker Caetano[3], Noboru Hioka[3], Kellen Brunaldi[1], Maria Ida Ravanelli[1*].

[1]*Departament of Physiological Sciences, State University of Maringa, Maringa, Parana, Brazil.*

[2]*Department of Chemistry, Federal University of Santa Catarina (UFSC), Florianópolis, SC, Brazil.*

[3]*Departament of Chemistry, State University of Maringa, Maringa, Parana, Brazil.*

[4]*Departament of Biochemistry, State University of Maringa, Maringa, Parana, Brazil.*

[5]*Department of Clinical Analysis and Biomedicine, State University of Maringa, Maringa, Parana, Brazil.*

[6]*Chemistry Graduation (COLIQ), Federal Technological University of Parana, Apucarana, Parana, Brazil.*

[7]*Department of Pharmacy and Pharmacology, State University of Maringa, Maringa, Parana, Brazil.*

Footnotes:

Camila Aparecida Errerias Fernandes Cardinali and Yandara Akamine Martins are currently affiliated with the Department of Physiology and Biophysics, University of São Paulo, São Paulo, Brazil.

Renato Sonchini Gonçalves is currently affiliated with the Center for Exact Sciences and Technologies (CCET), Federal University of Maranhão, São Luís, Maranhão, Brazil.

***Corresponding authors:**

Maria Ida Ravanelli

E-mail: mibrspeziali@uem.br

Phone number: +55 44 3011-4703

Camila Fabiano de Freitas Marin

E-mail: camila.f.freitas@ufsc.br

Phone number: +55 48 3721-4535

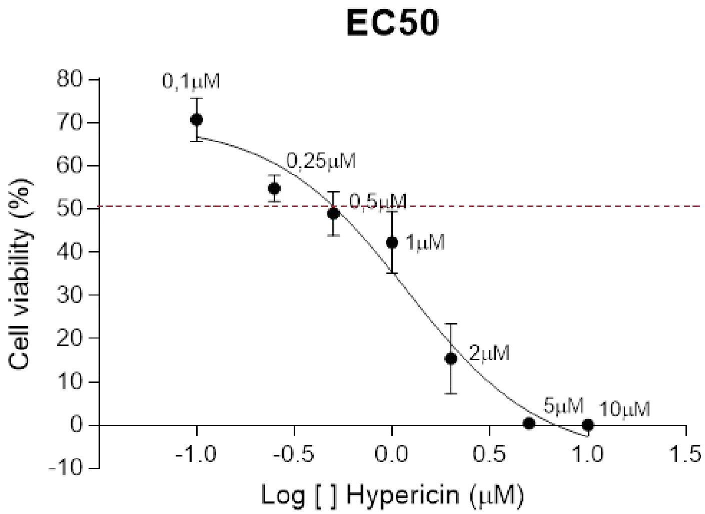

Figure 1S. EC50 of Hypericin in T98G cells (0.1, 0.25, 0.50, 1.00, 2.00, 5.00 and 10.00 μmol.L^{-1}) with an incubation time of 2hours. Lighting was carried out for 20' with 20 mW/cm^2 LED. Cell viability was determined using trypan blue dye after illumination. Cell survival was calculated by assuming total cells in the control well as 100% cell viability. Each value represents the MD±SEM of at least three experiments performed in triplicate.

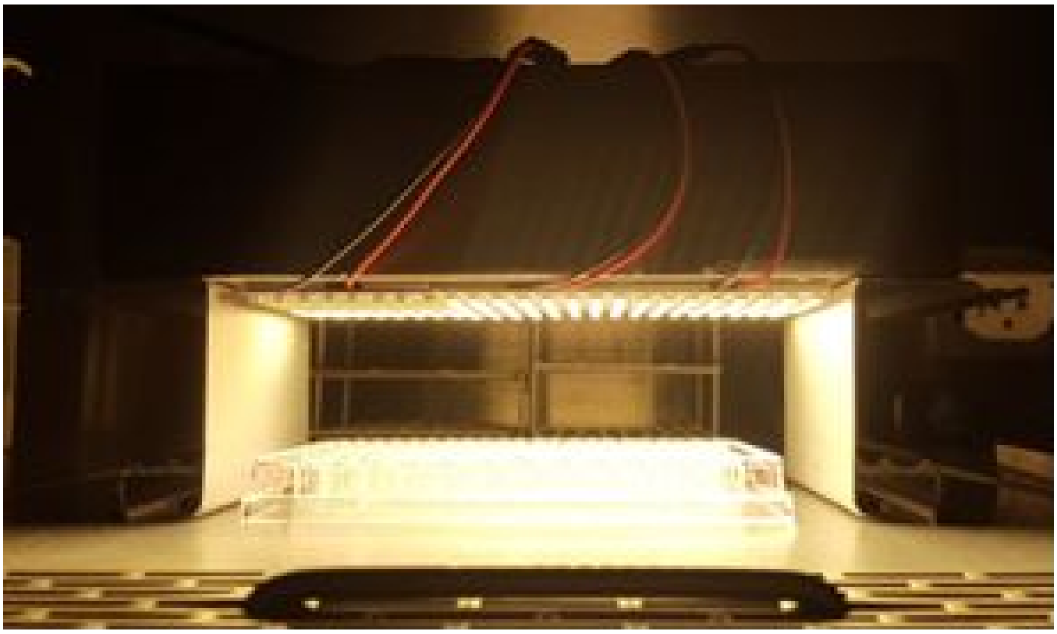

Figure 2S. Lighting system used in the present study.

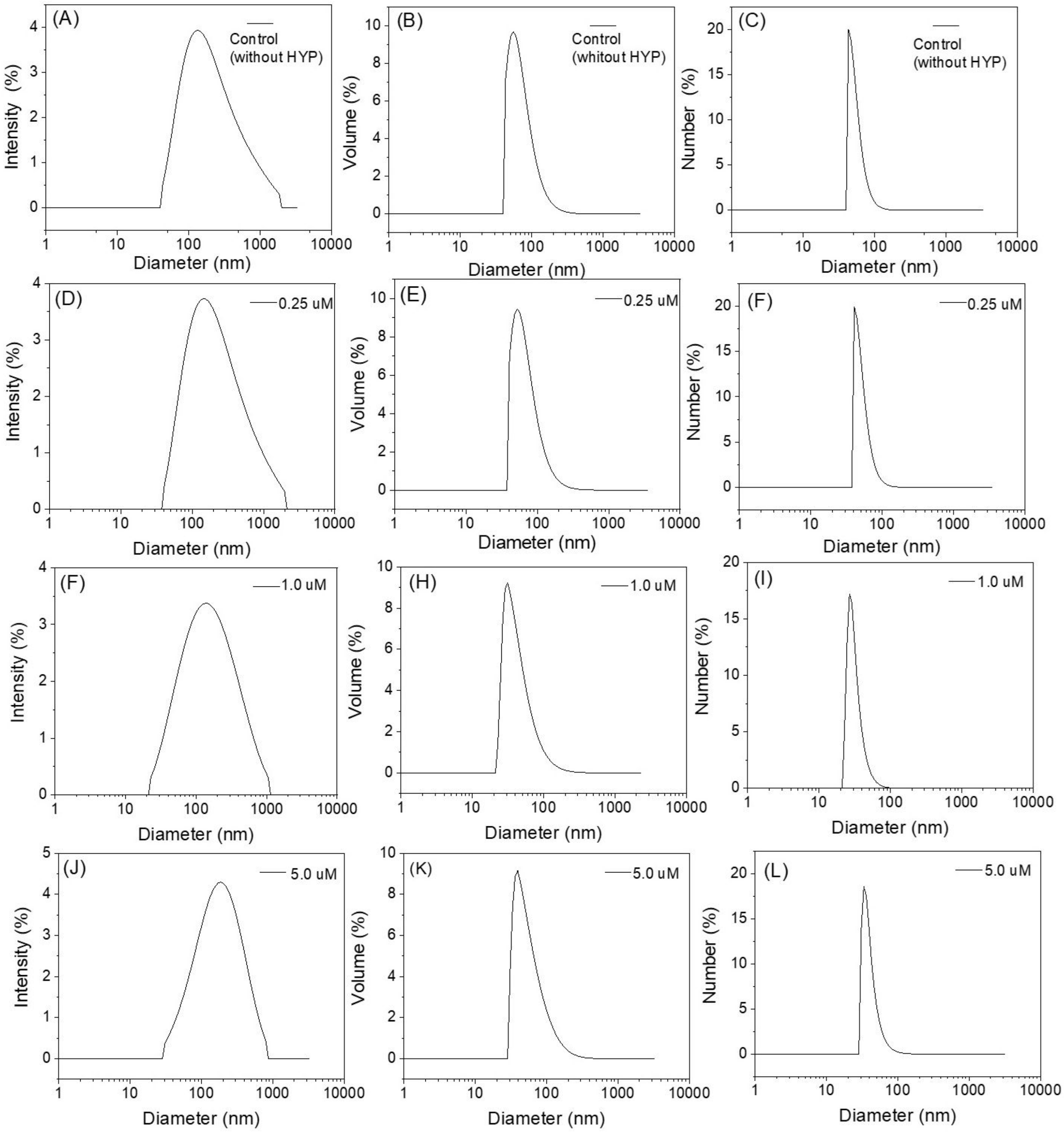

Figure 3S. Representative images from dynamic light scattering (DLS) analysis to evaluate the average size of liposomal vesicles based on intensity, volume, and number distribution for all vesicles produced, where A-C) represent the control liposome (without HYP), D-E) 0.25 μmol.L^{-1} of HYP; F-I) 1.00 μmol.L^{-1} of HYP; and J-L) 5.00 μmol.L^{-1} of HYP.
